# Verrucous carcinoma of the bladder with koilocytosis unassociated with vesical schistosomiasis

**DOI:** 10.1590/S1516-31802004000200006

**Published:** 2004-03-01

**Authors:** Fabio Lewin, Ana Paula Garcia Cardoso, Lucíla Heloísa Simardi, Marcos Tobias Machado

**Keywords:** Verrucous carcinoma, Bladder, Schistosomiasis, Human papillomavirus, Carcinoma verrucoso, Bexiga, Esquistossomose, Papiloma

## Abstract

**CONTEXT:**

Verrucous carcinoma of the bladder is a very rare malignant neoplasm, histologically similar to condyloma acuminatum. Usually it shows association with vesical schistosomiasis (bilharziasis). Only 13 cases unrelated to bilharziasis have been reported to date, and none of them reported koilocytosis, a typical finding in human papillomavirus infection.

**CASE REPORT:**

We report a case of verrucous carcinoma of the bladder that was unrelated to bilharziasis, with koilocytosis and absence of human papillomavirus. The literature relating to the topic is discussed.

## RESUMO

**CONTEXTO:**

O carcinoma verrucoso de bexiga é uma neoplasia maligna muito rara, histolo- gicamente semelhante ao condiloma acuminado. Normalmente apresenta-se associado à esquistossomose vesical (bilharzíase). Apenas 13 casos não relacionados à bilharzíase foram descritos até o momento, sendo que em nenhum deles foi relatada presença de coilocitose, um achado característico da in- fecção pelo papiloma vírus humano.

**RELATO DE CASO:**

Apresentamos um caso de carcinoma verrucoso de bexiga não relacionado à bilharzíase com presença de coilocitose e ausência de papiloma vírus humano. A literatura relacionada ao assunto é discutida.

## INTRODUCTION

Verrucous carcinoma of the bladder is a well-differentiated rare variant of squamous cell carcinoma. It is usually associated with vesical schistosomiasis (bilharziasis).^1^ Only 13 cases reported have shown no association with bilharziasis. Verrucous carcinoma spreads widely in the bladder and shows only local invasion.^2^ There is no reported case of human papillomavirus (HPV) infection in verrucous carcinoma of the bladder.

Our objective was to report on a case of verrucous carcinoma of the bladder with muscle invasion and koilocytosis (a typical finding in HPV infection), with discussion and review of the relevant literature.

## CASE REPORT

The patient was a 64-year-old white male, born and resident in São Paulo, Brazil, who was a smoker. He was interned with obstructive urinary symptoms and hematuria. Abdominal and pelvic computerized tomography showed a vesical tumor that apparently was not invasive, occupying 70% of the bladder, which was confirmed via cystoscopy. There were no signs of metastasis.

Histological examination of the transurethral resection diagnosed a verrucous squamous lesion with koilocytotic alterations, without invasion of the tunica muscularis.

The patient was subjected to radical cystoprostatectomy with urinary diversion. He did not present any postoperative inter-currence, and was discharged from hospital on the 15^th^ postoperative day.

**Figure 1:**
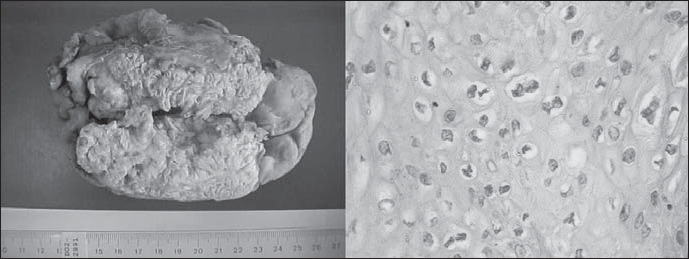
Macroscopic appearance: verrucous character of the neoplasm, involving the entire vesical wall (left). Photomicrograph: detail of the koilocytotic alterations (hematoxylin-eosin, 400 X).

Anatomopathological examination of the surgical material showed that the bladder contained an exophytic tumor measuring 9.5 × 7.5 × 7.0 cm, involving all of the walls (Figure 1). The cuts showed a thick white tumor that infiltrated the entire thickness of the wall and perforated it at the left side. There was no macroscopic infiltration of the prostate, seminal vesicles or urethral stumps, and the resected lymph nodes showed no metastasis.

Histological examination of the bladder showed an infiltrative verrucous carcinoma (pT3b) that involved the tunica muscularis and parts of the perivesical fibroadipose tissue. There were koilocytotic alterations (Figure 1), and the surgical margins did not show signs of neoplasm.

After three months of clinical follow-up, the disease returned with extensive involvement of the patient's pelvis. The patient subsequently died.

**Figure 2:**
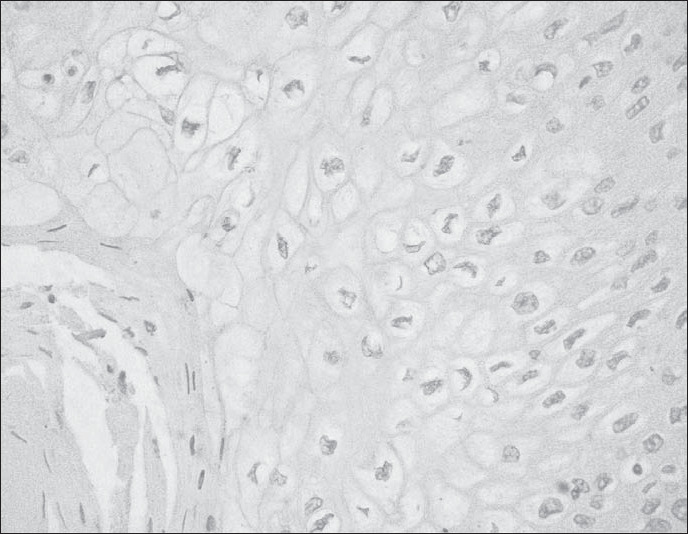
Photomicrograph of in situ hybridization, negative for human papillomavirus subtypes 6, 11, 16, 18, 30, 31, 33, 45, 51 and 52 (200 X).

Afterwards, *in situ* tests were performed to investigate hybridization (for HPV detection), immunohistochemistry (for detection of tumor suppression gene p53) and flux cytometry (to analyze DNA ploidy of the neoplastic cells). The *in situ* hybridization test was negative for the HPV subtypes 6, 11, 16, 18, 30, 31, 33, 35, 45, 51 and 52 (Figure 2). p53 was not detected in the immunohistochemical test. The flux cytometry yielded a DNA index of 1, which corresponds to a diploid DNA cellular population.

## DISCUSSION

Verrucous carcinoma occurs most frequently in the oral cavity, larynx, vagina, penis and perineum. Bladder occurrence is rare, and it is usually associated with vesical schis- tosomiasis.^3^ El-Sabai et al. studied 33 cases of verrucous carcinoma of the bladder and found an association with vesical schistosomiasis in 32 of them.^1^ Data from the literature shows that only 13 reported cases have been unrelated to bilharziasis. The etiology of verrucous carcinoma in these cases was not evident. The absence of bilharziasis as an endemic disease in Brazil and the findings from studies under the microscope ruled out vesical schistosomiasis from the present case.

Of all the cases described as verrucous carcinoma of the bladder that were unrelated to schistosomiasis, only three were analyzed for detection of HPV (by means of *in situ* hybridization), and that analysis was negative in all of the three cases. In that study, Cheng et al. compared those three cases with another three cases of condyloma acuminatum, making use of the same molecular analysis as applied in the present case. All of the three cases of condyloma acuminatum examined were positive for p53 and HPV, and the DNA was aneuploid. All of the three cases of verrucous carcinoma examined were positive for p53, in spite of the absence of HPV, and the DNA was also aneuploid.^4^ In our case, molecular analysis showed absence of p53 and HPV, with a diploid cell population.

It is well know that verrucous carcinoma of the bladder and condyloma acuminatum, including its aggressive variant of giant condyloma or Buschke-Löwenstein's tumor, have many histological similarities, which sometimes makes differential diagnosis very difficult. With regard specifically to Buschke-Löwenstein's tumor, there is an even greater resemblance to verrucous carcinoma. In addition to having the same histological characteristics, they also have similar clinical evolution, showing expansive growth that presses against the basal membrane.^5^ Human papillomavirus is the etiological agent for these condylomas. Pinto et al.^6^ identified only 17 cases of intravesical condyloma in reports in the literature.

In the present case, the koilocytotic alterations (typical of the cytopathic effect of human papillomavirus) may be suggestive of intravesical condyloma. However, the invasion of the tunica muscularis by the tumor (which is not a characteristic of condylomas) and the absence of human papillomavirus (as indicated by the *in situ* hybridization for the spectra of the examined viral subtypes) makes the diagnosis of verrucous carcinoma more probable. We must emphasize that the methodology applied shows only moderate sensitivity and specificity in comparison with other techniques for the detection of HPV, such as polymerase chain reaction (PCR), which shows very high sensitivity and specificity.

The present case shows many clinical-evolutive aspects similar to verrucous carcinoma of the bladder but unrelated to vesical schistosomiasis. However, the absence of HPV, as detected by *in situ* hybridization for the main subtypes, and especially the flux cytometry with diploid cells, make this a unique case. The presence of koilocytosis was an intriguing occurrence, since HPV was not detected in the lesion. Considering the rarity of the disease and the lack of molecular biology studies relating to it, other methods could be evaluated for characterizing such cases, in order to clarify the real origin of this pathological entity.
